# Photorefraction with Spot Vision Screener versus Visual Acuity Testing as Community-Based Preschool Vision Screening at the Age of 3.5 Years in Japan

**DOI:** 10.3390/ijerph19148655

**Published:** 2022-07-16

**Authors:** Toshihiko Matsuo, Chie Matsuo, Masami Kayano, Aya Mitsufuji, Chiyori Satou, Hiroaki Matsuoka

**Affiliations:** 1 Graduate School of Interdisciplinary Science and Engineering in Health Systems, Okayama University, Okayama 700-8558, Japan; matsuoc@okayama-u.ac.jp; 2 Department of Ophthalmology, Okayama University Hospital, Okayama 700-8558, Japan; 3 Okayama City Government Health Office, Okayama 700-8546, Japan; masami_kayano@city.okayama.lg.jp (M.K.); aya_mitsufuji@city.okayama.lg.jp (A.M.); chiyori_satou@city.okayama.lg.jp (C.S.); hiroaki_matsuoka@city.okayama.lg.jp (H.M.)

**Keywords:** strabismus, amblyopia, refractive error, photorefraction, preschool vision-screening program, Spot vision screener, visual acuity test, 3.5-year-old children, community health center, nurse

## Abstract

Nationwide in Japan, a community-based vision-screening program in 3.5-year-old children is conducted in three steps: questionnaires and home visual acuity testing as the primary screening; visual acuity testing by nurses and pediatricians’ inspection in community health centers as the secondary screening; and examinations by ophthalmologists as the tertiary screening. In this study, we introduced photorefraction with a Spot vision screener in addition to visual acuity testing to answer the clinical question of whether photorefraction could better detect eye diseases and potentially replace visual acuity testing. Photorefraction was performed on 813 consecutive 3.5-year-old children in a center. The children were sent to tertiary examinations, which were based on the Spot vision screener standard, in addition to the visual acuity testing standard: failure in either eye to pass 0.5 visual acuity in a center. A notice to visit ophthalmologists was issued for 95 children (11%), and documents with the diagnosis were sent back to the Heath Office for 76 children (80%). The rate of children with anisometropic or ametropic amblyopia or accommodative esotropia as treatment-requiring diseases was highest in cases of no pass at both standards (10/15 = 66%), and higher in cases of no pass only at the Spot vision screener standard (13/45 = 28%), compared with cases of no pass only at the visual acuity testing standard (6/33 = 18%, *p* = 0.0031). Photorefraction, in addition to visual acuity testing and inspection led to additional eye diseases detection at 3.5 years. Visual acuity testing at home would not be omitted in the introduction of photorefraction.

## 1. Introduction

Nationwide in Japan, developmental, physical, and mental checkups are carried out as a community-based health-screening program in all children at the age of 3 years, based on the Maternal and Child Health Act. The health-screening program also includes urinalysis, dental, visual, and hearing examinations. Historically, the 3-year-old health-screening program with dental checkup began in 1961, while the health-screening program for the 1.5-year-old children began in 1978, and dental checkup was added later in 1981. The visual and hearing examinations have been included as part of the checkups in children at the age of 1.5 years and 3 years since 1991 [1,2,3]. Each municipality can determine the exact month in the age of 3 years to invite children to Health Centers for their screening examinations. In Okayama Prefecture, in which the authors reside, 3-year-old screening examinations are conducted when children are at the age of 3.5 years, and thus, the term “3.5-year-old health-screening program” is used on some occasions.

The eye examinations as the vision-screening program for 3.5-year-old children in Japan are conducted in three steps [1,2,3]. As the primary screening, parents or guardians fill out questionnaires regarding specific problems such as squint and test the visual acuity of children using printed Landolt-C in two different sizes at home. As the secondary screening, nurses in Community Health Centers measure the uncorrected visual acuity in children who have neither undergone nor passed the visual acuity test at home, and pediatricians or medical officers inspect the eye condition, such as eye alignment. Orthoptists’ participation in this secondary screening process is not the standard in the checkups. As the tertiary screening, a notice is issued for children that are suspected of having diseases to visit ophthalmologists and undergo the eye examinations. The final diagnoses are sent back as documents to the City’s Health Office.

Autorefraction in preschool vision screening has been investigated for decades [4,5,6,7,8] to answer the clinical question of whether autorefraction is useful in detecting refractive errors that lead to the development of amblyopia. In our previous study, we conducted a prospective study, for the first time, to show the usefulness of autorefraction in the current system of visual acuity testing at home and in the Health Center [3]. A portable autorefractometer was used as the standard at the time that the study was conducted in 2007–2008. In the present study, we designed another prospective study to examine whether the introduction of photorefraction by the Spot vision screener [9,10,11,12,13,14,15,16,17] in addition to visual acuity testing in the current system would change the detection of eye diseases. A clinical question in this study is whether the Spot vision screener could replace visual acuity testing in the Health Centers from the viewpoint of sparing nurses’ workload.

## 2. Materials and Methods

### 2.1. Flow of 3.5-Year-Old Health-Screening Program

**Figure 1:**
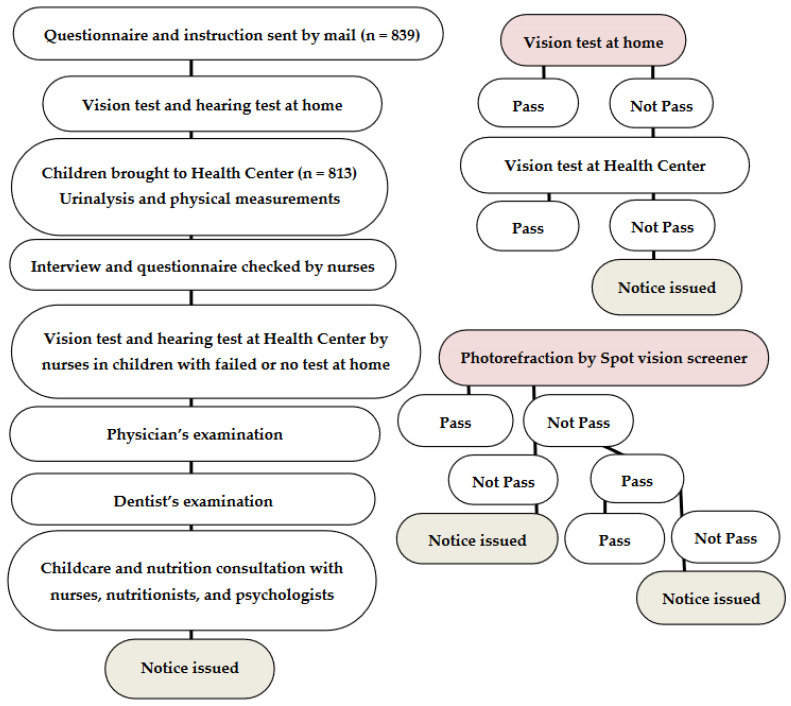
Flow chart of 3.5-year-old health-screening program (left column) and flow charts of visual acuity testing and photorefraction (right column) to issue a notice for the tertiary examination by ophthalmologists.

The population in Okayama City is about 700 thousand, and Okayama City Government Health Centers consist of 6 Community Health Centers: North-Ward-Central; North-Ward-North; Mid-Ward; East-Ward; South-Ward-West; and South-Ward-South. Each Community Health Center designates one day or two days in a month to the examination of children who have just turned 3.5 years old. In the flow of checkups (Figure 1), clinical laboratory technicians carry out urinalysis in children, while nurses check questionnaires, interview parents or guardians about the child’s past history, vaccination, daily sleeping and eating habits, and carry out physical measurements of children. Dentists examine children, and pediatricians or medical officers conduct developmental, physical, and mental assessments of children. Furthermore, nurses perform visual acuity tests and hearing tests in children who have either failed or have not undergone these tests at home.

### 2.2. Study Population

In this study, the Mid-Ward Community Health Center, with two days in a month for the examination, was chosen, and all 813 children who visited the Center on 16 consecutive days in 8 months from August 2020 to March 2021 were enrolled. Beforehand, invitations to visit this Health Center were sent to 839 eligible children in this period as a routine procedure. The total number of eligible children at 3.5 years old in Okayama City was 5943 from April 2020 to March 2021, and 5673 (95%) of these children visited health centers. This study was approved by the Ethics Committee of Okayama University Graduate School of Medicine, Dentistry, and Pharmaceutical Sciences, and Okayama University Hospital, as a collaborative study between the Okayama City Government Health Office and Okayama University (Identifier, 2005-002). The purpose of the study was explained in written form to parents or guardians who brought children to the Community Health Center, and informed consent was obtained from the parents or guardians.

### 2.3. Visual Acuity Testing at Home

A letter of notice to visit a Health Center is mailed in an envelope to each child, together with the instructions for the vision test, a vision questionnaire, and a sheet of paper on which large and small Landolt-C are printed on both sides: equivalent for visual acuity in decimals of 0.1 and 0.5, respectively, tested in the distance of 2.5 m. Parents or guardians first test the visual acuity of children with both eyes open, using the 0.1-equivalent Landolt-C at 1 m. The child’s visual acuity is then tested with the 0.5-equivalent Landolt-C in the distance of 2.5 m with both eyes open, and consequently in each eye when the other eye is covered with the examiner’s hand. Four directions of the Landolt-C (right, left, top, and bottom) are tested by rotating the printed paper, and the children pass the test when they correctly recognize at least three directions [2,3].

### 2.4. Questionnaire for Vision-Related Problems

The vision questionnaire (Table 1) asks whether visual acuity testing at home has been performed by parents or guardians, and whether 0.5-equivalent visual acuity was confirmed with both eyes open, and separately in each eye. The questions regarding eye-related problems are then asked: convergent, divergent, or vertical deviations; watching television in the near distance; abnormal head postures (head tilt, chin up or down, and face turn); winking outdoors; nystagmus; leukocoria; blepharoptosis; pupils of different sizes; eyelid fissure narrowing; and slow movement in the dark. Any eye diseases that have been diagnosed by ophthalmologists are also questioned [2,3].

### 2.5. Visual Acuity Testing and Spot Vision Screener in Health Center

In the Community Health Center, nurses test the visual acuity in each eye of children who have either failed or have not undergone the visual acuity testing at home (Figure 1): 0.1 and 0.5-equivalent Landolt-C cards are shown in the distance of 5 m. The children pass the test when they correctly recognize at least three directions of the 0.5-equivalent Landolt-C. Photorefraction with the Spot vision screener (Welch Allyn, Skaneateles Falls, NY, USA) in the present study was performed in all children by one examiner (C.M.). At the final step, all the children are inspected systemically by pediatricians or medical officers.

Regarding the hearing checkup, the invitation that is mailed to each child also contains the instructions for the hearing test, a hearing questionnaire, and a sheet of paper, which is printed with 6 figures such as a dog, cat, elephant, chair, umbrella, and ear. Parents or guardians fill in the questionnaire for hearing problems and test the hearing of children by whispering 6 words for the printed figures with their mouth covered, and while at a distance of 1 m from the child. Children pass the test when they point out the correct figures with their fingers. Nurses in the Community Health Center repeat the hearing test for children who have either failed or have not undergone the test at home.

### 2.6. Criteria to Issue a Notice for the Tertiary Examinations

A notice to visit ophthalmologists is issued to children who have problems that are raised by the questionnaire or who have failed the visual acuity testing in the Health Centers, or who are pointed out to have problems by pediatricians or medical officers. In addition to these current criteria, a notice was issued in this study by photorefractive criteria (manufacturer’s criteria for the age of 36–72 months) [9]: greater than 1.0-diopter in the difference of spherical equivalents between the right eye and left eye for anisometropia, greater than 1.25-diopter spherical power for myopia in either eye, greater than 2.5-diopter spherical power for hyperopia in either eye, and/or greater than 1.75-diopter cylindrical power for astigmatism in either eye, greater than 1 mm in the difference of pupil diameters between the right eye and left eye for anisocoria, greater than 5 degrees in esodeviation or 8 degrees in exodeviation for strabismus. Photorefraction was finished with the first measurement indicated as normal. When the first measurement was indicated as abnormal, the second measurement was carried out: two successive measurements that were indicated as abnormal were finished and labeled as abnormal, while the second measurement that was indicated as normal led to the third measurement. When the second and third measurements were indicated as normal, the result was labeled as normal. When the third measurement was indicated as abnormal, it was then finally labeled as abnormal (Figure 1). Photorefraction could be measured in all 813 children in this study. The final diagnoses in the documents that were filled in by ophthalmologists and sent back to the Okayama City Government Health Office were reviewed with respect to the current criteria (visual acuity testing at home or by nurses, questionnaire, and doctors’ inspection) versus the photorefractive criteria.

### 2.7. Primary Outcome and Statistical Analyses

A chi-square test in a 2 × 3 table was used to compare the outcome among three groups of children: no pass only at the Spot vision screener standard, no pass only at the visual acuity testing standard, and no pass at both standards as the reasons for being issued a notice to visit ophthalmologists. The primary outcome was the number (the rate) of children with a diagnosis of anisometropic or ametropic amblyopia or accommodative esotropia as treatment-requiring diseases. The secondary outcome was the number (the rate) of children with the notice who visited ophthalmologists to have the final diagnosis sent back to the City’s Health Office.

The spherical equivalents, spherical powers, and cylindrical powers of refractive errors in the right eye and left eye that were measured by the Spot vision screener were correlated between both eyes in each child by a Spearman rank correlation coefficient. The mean (95% confidence interval) and the standard deviation were calculated for the spherical equivalents, spherical powers, and cylindrical powers in the right eye and left eye.

## 3. Results

### 3.1. Visual Acuity Testing and Spot Vision Screener

Of the 813 total children, nurses in the Health Center tested visual acuity in 73 children who did not undergo visual acuity tests or did not pass visual acuity testing at home. Of these 73, 10 children passed the visual acuity of 0.5 in each eye (Table 2), while the remaining 63 children could not pass the visual acuity test (Table 3). In contrast, 71 children did not pass the Spot vision screener standard (Table 3 and Table 4). A notice to visit ophthalmologists for the detailed examination as the tertiary screening was issued for 95 (11%) of 813 children, and 76 (80%) of those visited the ophthalmologist so that their final diagnoses could be sent back to the City’s Health Office.

Of the 95 children with a notice issuance, 15 children passed neither the Spot vision screener standard nor the visual acuity testing standard; 45 children did not pass the Spot vision screener standard and did pass the visual acuity testing standard; while 33 children did pass the Spot vision screener standard and did not pass the visual acuity testing standard (Table 3). A notice to visit ophthalmologists was issued because of other reasons in the remaining two children who passed both the Spot vision screener standard and the visual acuity testing standard. A notice was required to be issued by the procedural protocol, but this was not issued to the other 21 children who did not pass either standard or both standards, mainly because they were currently followed by ophthalmologists. The rates of children who visited ophthalmologists and had their final diagnoses sent back to the Health Office were 80% (12 of 15 children) in the group with no pass at both standards; 93% (42 of 45 children) in the group with no pass only at the Spot vision screening standard; and 60% (20 of 33 children) in the group with no pass only at the visual acuity testing standard. The rates were significantly different among the three groups (*p* = 0.0018, chi-square test).

### 3.2. Diagnoses in the Tertiary Examinations by Ophthalmologists

The overall diagnoses which were reported back from ophthalmologists were anisometropic or ametropic amblyopia, accommodative esotropia, intermittent exotropia, and refractive errors such as hyperopia, hyperopic, mixed, and myopic astigmatism (Table 3). Rare diagnoses were anisocoria, entropion, and Down syndrome, which were also associated with a kind of refractive error in some children. Among the children with a notice issued to visit ophthalmologists, the rate of children with anisometropic or ametropic amblyopia or accommodative esotropia as treatment-requiring diseases was highest in the group with no pass at both standards (10/15 = 66%), and higher in the group with no pass at the Spot vision screener standard and a pass at the visual acuity testing standard (13/45 = 28%), compared with the rate in the group with a pass at the Spot vision screener standard and no pass at the visual acuity testing standard (6/33 = 18%). The detection of treatment-requiring diseases was significantly different among the three groups (*p* = 0.0031, chi-square test).

### 3.3. Measurements by Spot Vision Screener

**Figure 2:**
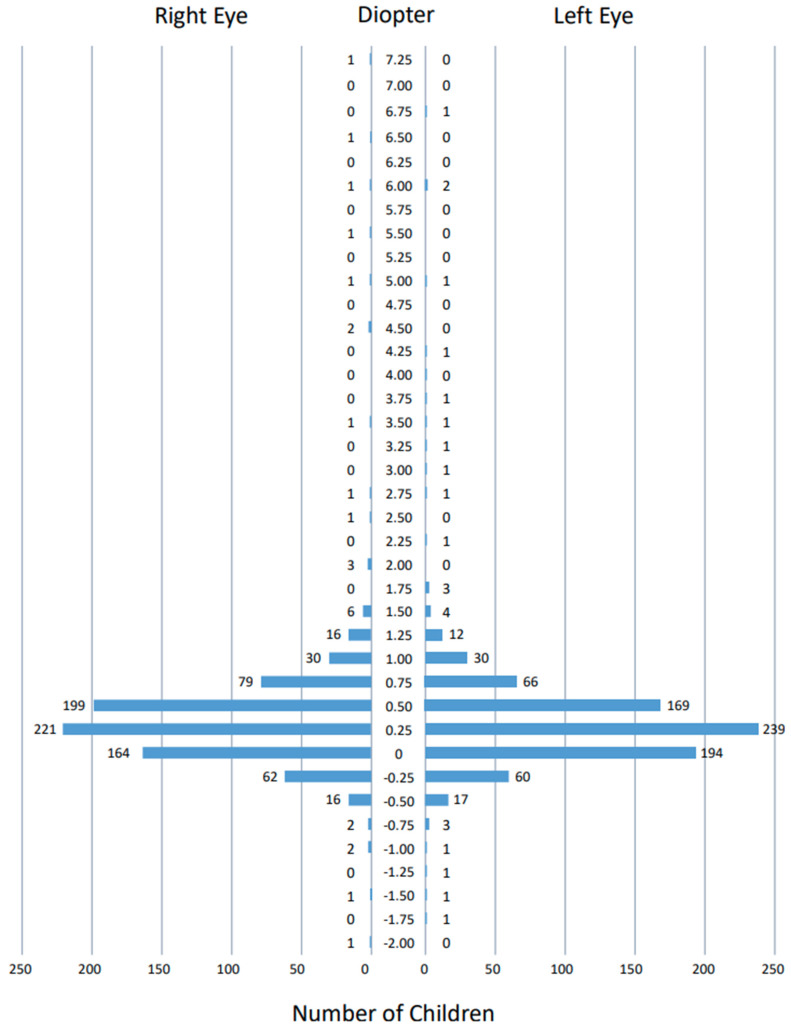
Distribution of spherical equivalents of refractive errors measured by Spot vision screener in 813 right eyes and 813 left eyes of all 813 children. Two measurements with a value indicated as >+7.5 diopters in the right eye of a child and the left eye of another child are not plotted.

**Figure 3:**
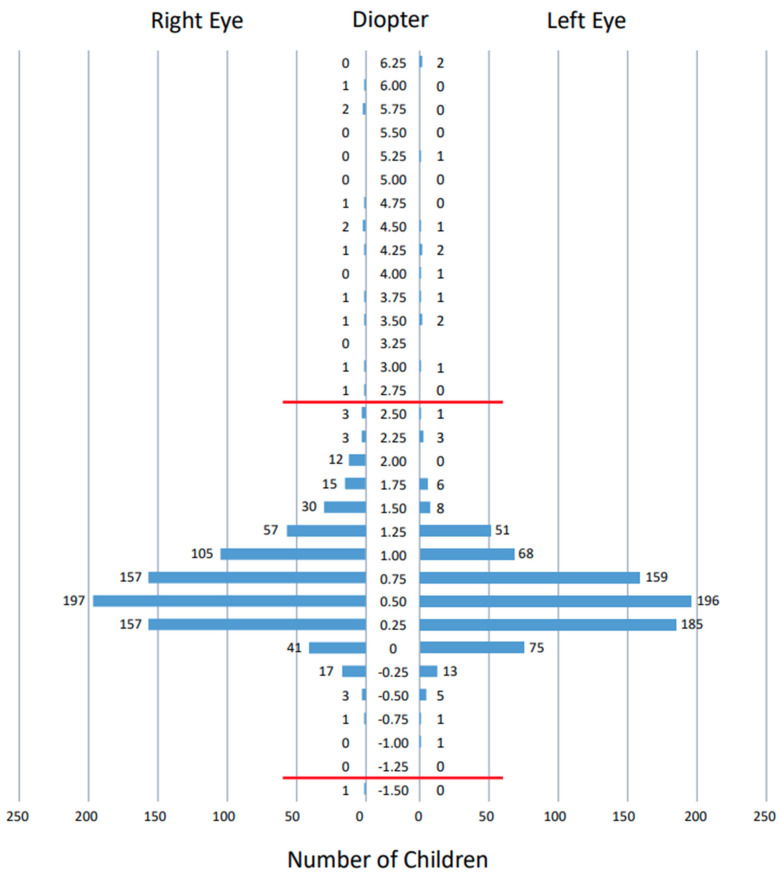
Distribution of spherical powers of refractive errors measured by Spot vision screener in 813 right eyes and 813 left eyes of all 813 children. Five measurements with a value indicated as >+7.5 diopters in both eyes of two children and the right eye of another child are not plotted. Red horizontal lines indicate borderlines outside the normal range set by Spot vision screener at the age of 36–72 months: hyperopia greater than +2.5 diopters and myopia greater than −1.25 diopters.

**Figure 4:**
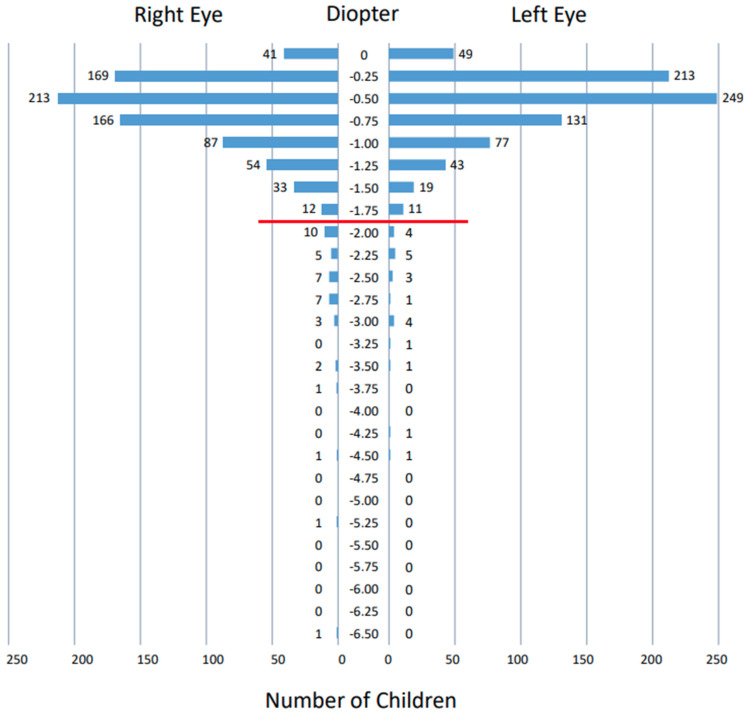
Distribution of cylindrical powers of refractive errors measured by Spot vision screener in 813 right eyes and 813 left eyes of all 813 children. Red horizontal line indicates the borderline outside the normal range set by Spot vision screener at the age of 36–72 months: astigmatism greater than −1.75 diopters.

The spherical equivalents (Figure 2), spherical powers (Figure 3), and cylindrical powers (Figure 4) of refractive errors were measured successfully by the Spot vision screener in both eyes of all 813 children that were involved in this study. The spherical equivalents of refractive errors were distributed predominantly in the range of –0.5 to +1.5 diopters in both eyes (Figure 2). The spherical powers in refractive errors were distributed predominantly in the range of –0.5 to +2.0 diopters in both eyes (Figure 3), while the cylindrical powers were distributed predominantly in the range of –2.0 to 0 diopters in both eyes (Figure 4). The mean (95% confidence interval) and the standard deviation of the spherical equivalents (N = 813) were 0.37 (0.32–0.42) diopters and 0.70 diopters in the right eye and 0.33 (0.28–0.38) diopters and 0.66 diopters in the left eye. The mean (95% confidence interval) and the standard deviation of the spherical powers (N = 813) were 0.74 (0.68–0.79) diopters and 0.77 diopters in the right eye and 0.65 (0.60–0.70) diopters and 0.73 diopters in the left eye. The mean (95% confidence interval) and the standard deviation of the cylindrical powers (N = 813) were −0.62 (−0.66 to −0.59) diopters and 0.51 diopters in the right eye and −0.74 (−0.78 to −0.69) diopters and 0.61 diopters in the left eye. The refractive errors had a statistically significant correlation when compared between both eyes (*p* < 0.0001 for spherical equivalents, *p* < 0.0001 for spherical powers, and *p* < 0.0001 for cylindrical powers, Spearman rank correlation coefficient).

Table S1 shows the measurements of the Spot vision screener in 43 children who passed the Spot vision screener standard and did not pass the visual acuity testing standard. All these values within the normal range that was set by the Spot vision screener were not so deviated, compared with the overall distribution, even in children who were pointed out to have treatment-requiring diseases such as anisometropic and ametropic amblyopia in the tertiary examinations by ophthalmologists.

## 4. Discussion

In Japan, vision-screening programs in children and students are conducted as part of health-screening programs which include developmental, physical, and mental checkups, in combination with dental checkup and hearing-screening programs. Children have examinations at 1.5 and 3.5 years, and at 5 years, just before they attend elementary school. Students at each grade of elementary school, junior high school, and high school, from 6 to 17 years of the age, undergo visual acuity testing every year at school [18,19]. Visual acuity testing is also recommended to be performed in kindergarten. Visual acuity testing at school age is mainly aimed at detecting refractive errors, especially myopia, which is at a higher rate in Japan [20]. The vision-screening program in the earlier years of life is also intended to detect rare congenital diseases such as congenital cataract [21], ectopia lentis [22], ocular surface dermoid lesions [23], congenital ptosis [24], and congenital superior oblique muscle palsy [25]. These rare diseases are frequently associated with refractive errors that lead to the development of amblyopia. The vision questionnaire in the current screening system for 3.5-year-old children (Table 1) is designed to detect these rare eye diseases, while visual acuity testing is performed to detect refractive errors that lead to amblyopia [2,3].

We previously designed a prospective study to answer the clinical question of whether the addition of autorefraction in the long-lasting current system with visual acuity testing since 1991 would lead to the better detection of eye problems in the preschool vision-screening program for 3.5-year-old children [3]. The introduction of autorefraction requires at least one additional examiner and takes longer in the whole process of the 3.5-year-old health-screening program. Furthermore, nurses have to perform visual acuity tests and hearing tests for children who have either failed or have not undergone these tests at home. All the costs of the 3.5-year-old examinations, including the cost of the tertiary examination that is performed by ophthalmologists, are paid by the City’s Health Office, namely, the taxpayers’ money. In a previous study involving 265 children [3], ametropic amblyopia in one child was detected by the addition of autorefraction, while a large number of children with refractive errors which were mostly designated as not serious were also detected by autorefraction.

In contrast to autorefraction with a hand-held autorefractometer in our previous study [3], photorefraction with the Spot vision screener in the present study led to the detection of a smaller percentage of children with no pass at the refractive standard. Thus, the total number of notices that were issued for the tertiary examination became as low as 11% (95/813) in the present study, compared with 24% (64/265) in our previous study [3]. Of these children with a notice issuance for the tertiary examination, the notice was issued to 45 children (5%) based on photorefractive criteria only versus 50 children (6%) based on visual acuity testing and other criteria in the present study, while the notice was issued to 42 children (16%) with autorefractive criteria only versus 22 children (8%) based on visual acuity testing and other criteria in our previous study [3]. The time that was required for the measurement was apparently shorter with the Spot vision screener in the present study than with a hand-held autorefractometer in our previous study. It should be noted that in the present study, six children with anisometropic or ametropic amblyopia as treatment-requiring diseases were found in the group with a pass at the Spot vision screener standard and no pass at the visual acuity testing standard. Regarding the clinical question in this study, visual acuity testing did indeed detect treatment-requiring diseases which were not detected by photorefraction, and thus, the visual acuity testing would not be omitted in the 3.5-year-old vision-screening program.

The present study had a major limitation in that 19 children did not appear to undergo the tertiary examinations by ophthalmologists after a notice was issued for them to organize a visit. The rate of no visit to ophthalmologists was especially high in the group with a pass at the Spot vision screener standard and no pass at the visual acuity testing standard (13/33 = 39%), compared with the group with no pass at both standards (3/15 = 20%) and the group with no pass at the Spot vision screener standard and a pass at the visual acuity testing standard (3/45 = 6%). These facts might be explained by the feeling of parents or guardians that the visual acuity testing that is performed at home or in the Health Center appears to be unreliable when compared with the numerical results of photorefraction.

Another limitation of this study would be the fact that the tertiary examinations were performed by many different ophthalmologists in Okayama City. These ophthalmologists are all certified by the Board of Ophthalmology in Japan and follow the standard of care in the general field of ophthalmology. Pediatric patients who are suspected of amblyopia are examined by cycloplegic refraction, which means refractive testing by a table-fixed or hand-held autorefractometer under cycloplegia with topical 1% cyclopentolate. In case of esotropia, cycloplegic refraction with topical 0.5% or 1% atropine for 3–5 days is recommended [26,27]. Based on the cycloplegic refraction, glasses with full correction are prescribed as a standard treatment for amblyopia. Even within the standard treatment, small differences in the interpretation may exist among ophthalmologists regarding the borderline of refractive errors to prescribe glasses.

The photorefractive criteria for sending 3.5-year-old children to the tertiary examinations by ophthalmologists in this study was based on the manufacturer’s setting [9]. To check the clinical validity of this setting, we depict as graphs (Figure 2, Figure 3 and Figure 4) the distribution of spherical equivalents, spherical powers, and cylindrical powers of refractive errors in all 813 children who joined this study. In other words, we try to answer another clinical question: whether the borderline for the normal range that is set by the Spot vision screener is reasonable. We assume that the spherical and cylindrical powers of refractive errors might be somewhat deviated, even within the normal range in children who did pass the photorefractive criteria but did not pass the visual acuity testing standard. Against our expectations, the spherical and cylindrical powers of refractive errors in these 43 children are not so deviated within the normal range (Table S1), leading to the conclusion that there is no evidence so far to change the borderline that is set by the Spot vision screener.

Since the line of examinations and the time schedule for photorefraction were evaluated in the present trial in the fiscal year 2020, in the fiscal year 2021 starting from April 2021, photorefraction with the Spot vision screener was introduced as the vision-screening program in all Community Health Centers in Okayama City to cover all children at the age of 3.5 years. In each Community Health Center, one nurse is in charge of photorefraction, while a few nurses offer extra help. Visual acuity testing at home is continued, while visual acuity testing in Community Health Centers by nurses on children with failed tests or no testing at home is discontinued due to the shortage of personnel. Instead, parents or guardians who accompany the children are asked to test the visual acuity of children in Community Health Centers in the case that visual acuity testing has not been performed at home. The criteria to issue a notice for the tertiary examinations by ophthalmologists are the same as in this study by the visual acuity testing standard and the Spot vision screener standard. We plan to review the real-world data on the 3.5-year-old vision-screening program in the fiscal year 2021 to check whether the new system with photorefraction detects treatment-requiring diseases such as anisometropic and ametropic amblyopia.

## 5. Conclusions

Photorefraction with the Spot vision screener was introduced preliminarily on the current line of visual acuity testing at home and in the Health Center as the vision-screening program that was part of the 3.5-year-old health-screening program. Based on the notice that was issued for children to have the tertiary examinations by ophthalmologists, returned documents to the City’s Health Office were analyzed from the viewpoint of the reasons for issuance: pass or no pass at the visual acuity testing standard or the Spot vision screener standard. Photorefraction did detect treatment-requiring eye diseases such as anisometropic and ametropic amblyopia, which were otherwise not detected by visual acuity testing at home and in the Health Center. On the contrary, it should be noted that several children were reported to have anisometropic and ametropic amblyopia, which were detected only by the visual acuity testing standard, and not by the Spot vision screening standard. Therefore, with the introduction of photorefraction, visual acuity testing would not be eliminated in the 3.5-year-old vision-screening program.

Another study for the vision-screening program in 294 children at 3.5 years old, carried out concurrently in a different city of Japan, reached the same conclusion as in the present study [16]. Based on the long-term experience of the nationwide vision-screening program, which has been conducted since 1991 in Japan, we can convey the following message to policy makers and professionals in this country and other countries: photorefraction by the Spot vision screener is effective in detecting amblyogenic conditions, but the Spot vision screener alone as a vision-screening method is unsuitable for detecting all amblyogenic risk factors in children at 3.5 years old. It is desirable, in parallel, to conduct visual acuity testing at home, which has been routinely performed as the vision-screening program in Japan [1,2,3].

## Figures and Tables

**Table 1:** Vision questionnaire sent to children at 3.5 years old.

Vision Test at Home	
Did you do the vision test?	Yes or No
2. Did the child understand and complete the vision test?	Yes or No
3. Did the child see the direction of small C with both eyes open?	Yes or No
4. Did the child see the direction of small C with the right eye only?	Yes or No
5. Did the child see the direction of small C with the left eye only?	Yes or No
Questions for symptoms	
Do the child’s eyes show inward deviation?	Yes or No
2. Do the child’s eyes show outward or upward deviation?	Yes or No
3. Does the child watch television in the near distance and appear to have difficulty seeing in the far distance?	Yes or No
4. Does the child have the tendency in seeing?	
(1) Frown or narrow the eyes	Yes or No
(2) Tilt the head	Yes or No
(3) Turn the face and cast a side glance	Yes or No
(4) Drop the chin and glance up	Yes or No
5. Does the child close one eye in bright outdoors?	Yes or No
6. Are the child’s eyelids droopy?	Yes or No
7. Are the child’s eyes shaky when staring?	Yes or No
8. Does the child move slowly in the dark?	Yes or No
9. Do the child’s pupils appear whitish?	Yes or No
10. Do the child’s pupils appear different in size?	Yes or No
11. Does the child show drooping eyes and chin-up position in the evening, but not in the morning?	Yes or No
12. Has or is the child being seen by an eye doctor? What is the diagnosis?	Yes or No
13. Do you have any question regarding the child’s eyes?	

**Table 2:** Reasons for failure in visual acuity testing standard.

Visual Acuity Testing Results	No Pass at Spot Vision Screener Standard	Pass at Spot Vision Screener Standard	Total
Pass at testing in Health Center	10	0	10
Failure in Health Center (in total)	20	43	63
0.5 in the right eye, 0.1 in the left eye	2	1	3
0.1 in the right eye, 0.5 in the left eye	2	3	5
0.1 in both eyes	10	7	17
Unmeasurable in both eyes	6	32	38

**Table 3:** Issuance of a notice to visit ophthalmologists based on visual acuity testing standard and Spot vision screener standard in 95 children out of 813 children.

Reasons for Issuance			Notice							Diagnoses on Returned Documents	
Visual acuity testing standard	Spot vision screener standard	Others	Required but not issued	Reasons for non-issuance		Issued in total	Issued and retuned with no findings	Issued and returned with findings	Issued but not returned		
Pass	No pass	none	6	Followed	6	45	4	38 (12 *)	3	Anisometropic amblyopia Ametropic amblyopia Accommodative esotropia Intermittent exotropia Hyperopia/hyperopic astigmatism Mixed astigmatism Myopic astigmatism Anisocoria Entropion	4 8 1 3 13 6 3 1 1
No pass	No pass	none	5	Followed	5	15	0	12 (6 *)	3	Anisometropic amblyopia Ametropic amblyopia Accommodative esotropia Hyperopia/hyperopic astigmatism Mixed astigmatism Myopic astigmatism Down syndrome	5 4 1 1 1 1 1
No pass	Pass	none	10	Followed No wish Unknown	5 1 4	33	7	13 (2 *)	13	Anisometropic amblyopia Ametropic amblyopia Intermittent exotropia Hyperopia/hyperopic astigmatism Mixed astigmatism Myopic astigmatism	2 4 2 2 1 1
Pass	Pass	photophobia	0			2	2	0	0		
In total			21			95	13	63	19		

“Followed” indicates “Followed by ophthalmologists”. (numerals *) indicate the number of children with the statement on the returned documents that treatment is required. Diagnoses in rightmost column overlap in some children.

**Table 4:** Reasons for failure in Spot vision screener standard.

	Pass at Visual Acuity Testing Standard	No pass at Visual Acuity Testing Standard	Total
Anisometropia, astigmatism	2	0	2
Anisometropia	4	1	5
Anisometropia, hyperopia	4	6	10
Anisometropia, hyperopic astigmatism	0	0	0
Astigmatism in either eye	18	4	22
Astigmatism in both eyes	16	4	20
Hyperopia in either eye	0	0	0
Hyperopia in both eyes	4	2	6
Myopia in both eyes	0	2	2
Strabismus	8	3	11
Anisocoria	1	0	1
In total	57	22	79

Reasons overlap in some children.

## Data Availability

The original data sheet, created and analyzed in the current study, is available from the corresponding author on reasonable request.

## Supplementary Materials

The following supporting information can be downloaded at: https://www.mdpi.com/article/10.3390/ijerph19148655/s1, Table S1: Measurements of Spot vision screener in 43 children who passed Spot vision screener standard and did not pass visual acuity testing standard.
