# Supplementary material for: Photorefraction with Spot Vision Screener versus Visual Acuity Testing as Community-Based Preschool Vision Screening at the Age of 3.5 Years in Japan
Source: Int J Environ Res Public Health. 2022 Jul 16;19(14):8655. doi: 10.3390/ijerph19148655 (PMC9321534; doi:10.3390/ijerph19148655)
Supplement: Supplementary file 1 [file ijerph-19-08655-s001.zip › ijerph-1785545-supplementary.pdf]

Table S1. Measurements of Spot vision screener in 43 children who passed Spot vision screener standard and did not pass visual acuity-testing standard

No.	Spot vision screener measurement (diopter)						Visual acuity testing	Tertiary examination		Reported diagnosis
	Right Eye			Left Eye				Issuance	Visit	
	SE	DS	DC	SE	DS	DC				
1	0	+0.5	-1.25	0	+0.5	-0.75	Unable	Yes	No	
2	+0.75	+1.0	-0.75	+0.5	+1.0	-1.0	Unable	Yes	Yes	Unable to examine
3	+0.5	+1.25	-1.5	+1.25	+1.75	-1.25	Unable	Yes	Yes	No findings
4	+0.25	+0.75	-0.75	0	+0.25	-0.5	Unable	Yes	Yes	No findings
5	+0.25	+0.5	-0.5	+0.25	+0.5	-0.5	Unable	Yes	Yes	Intermittent exotropia
6	0	+0.25	-0.5	+0.25	+0.75	-0.75	Unable	No	No	
7	+0.25	+0.75	-1.0	+0.25	+0.75	-1.0	Unable	No	No	
8	+0.25	+0.5	-0.5	0	0	-0.25	Unable	No	No	
9	+0.5	+0.75	-0.5	0	+0.25	-0.5	0.1, both eyes	Yes	No	
10	+0.5	+0.5	-0.5	+0.25	+0.5	-0.5	Unable	Yes	Yes	No findings
11	+0.25	+1.0	-1.5	-0.25	0	-0.5	0.1, right eye	Yes	Yes	No findings
12	0	0	-0.25	0	+0.25	-0.5	Unable	Yes	Yes	Hyperopia
13	-0.25	+0.25	-1.0	-0.25	0	-0.25	0.1, both eyes	Yes	No	
14	+0.75	+1.25	-0.75	+0.75	+1.0	-0.75	Unable	No	No	
15	-0.25	+0.25	-1.25	-0.5	-0.25	0	Unable	Yes	No	
16	-0.25	0	-0.25	-0.25	+0.25	-0.75	0.1, both eyes	Yes	Yes	Ametropic amblyopia
17	+0.5	+0.75	-0.5	+0.5	+0.75	-0.5	Unable	Yes	Yes	No findings
18	-0.25	0	-0.5	+0.25	+0.5	-0.5	0.1, both eyes	Yes	No	
19	-0.5	-0.5	0	-0.5	0	-0.5	Unable	No	No	
20	-0.25	+0.25	-0.75	0	+0.25	-0.5	Unable	Yes	Yes	Mixed astigmatism
21	-0.5	-0.25	-0.5	-0.5	-0.25	-0.5	Unable	Yes	No	
22	+0.25	+0.5	-0.5	+0.25	+0.5	-0.25	Unable	Yes	No	
23	+0.5	+0.5	0	+0.5	+0.75	-0.5	Unable	No	No	
24	+0.25	+0.5	-0.5	0	0	-0.25	Unable	Yes	Yes	Anisometropic amblyopia
25	+0.5	+0.75	-0.5	+0.5	+0.5	0	Unable	No	No	
26	-0.5	+0.25	-1.25	-0.25	0	-0.75	0.1, right eye	Yes	Yes	Intermittent exotropia
27	+0.5	+1.0	-1.0	+0.75	+1.0	-0.5	Unable	Yes	Yes	Hyperopia
28	-0.25	0	0	+0.25	+0.5	-0.25	0.1, right eye	Yes	Yes	Ametropic amblyopia
29	0	+0.25	-0.5	0	0	0	0.1, both eyes	Yes	No	

30	+0.25	+0.5	-0.5	+0.5	+1.25	-1.25	Unable	No	No	
31	+0.25	+0.75	-1.0	+0.5	+0.75	-0.25	0.1, both eyes	Yes	Yes	Ametropic amblyopia
32	+0.25	+0.75	-1.0	+0.25	+0.5	-0.5	Unable	Yes	No	
33	0	+0.25	-0.25	0	0	-0.25	Unable	Yes	No	
34	+0.25	+1.0	-1.5	+0.5	+1.25	-1.5	Unable	Yes	No	
35	+0.25	+0.25	0	0	+0.25	-0.5	Unable	Yes	Yes	No findings
36	+0.75	+1.0	-0.75	+0.5	+0.75	0	Unable	No	No	
37	-0.5	-0.25	-1.0	-1.0	-0.5	-1.0	Unable	Yes	Yes	Anisometropic amblyopia
38	+0.25	+0.5	-0.25	+0.25	+0.5	-0.25	Unable	Yes	Yes	Ametropic amblyopia
39	+1.0	+1.75	-1.5	+0.75	+1.25	-1.0	Unable	Yes	No	
40	+1.0	+1.25	-0.5	+0.75	+1.0	-0.5	Unable	No	No	
41	+0.5	+0.75	-0.5	+0.5	+0.5	-0.25	0.1, both eyes	Yes	Yes	Myopic astigmatism
42	0	+0.25	-0.25	0	+0.25	-0.25	Unable	Yes	Yes	No findings
43	+0.25	+0.5	-0.5	+0.75	+1.0	-0.75	0.1, left eye	Yes	No	

SE, spherical equivalent of refractive error; DS, degree of spherical power of refractive error; DC, degree of cylindrical power of refractive error
